# Influence of Temperature on Characteristics of Particulate Matter and Ecological Risk Assessment of Heavy Metals during Sewage Sludge Pyrolysis

**DOI:** 10.3390/ma14195838

**Published:** 2021-10-06

**Authors:** Binbin Li, Haihong Fan, Songxiong Ding, Yixuan Luan, Yiming Sun

**Affiliations:** 1 College of Materials Science and Engineering, Xi’an University of Architecture and Technology, Xi’an 710055, China; libinbin1987@xauat.edu.cn (B.L.); luanyixuan2020@163.com (Y.L.); xajzkj2020tzzdx@163.com (Y.S.); 2 Faculty of Science and Engineering, University of Agder, N4879 Grimstad, Norway

**Keywords:** sewage sludge, particulate matter, heavy metals, ecological risk assessment

## Abstract

The formation process of Particulate Matter (PM) during sludge pyrolysis at different temperatures (300–700 °C) and the ecological risks of heavy metals were studied. The results showed that the particulate matter is mainly condensed on the quartz film in a carbon-based organic matter when the pyrolysis temperature was between 200–500 °C in a volatilization process. Inorganic particles was found in the particulate matter when the temperature was raised to 500–700 °C in a decomposition stage. Heavy metals were enriched in particulate matter with increase in pyrolysis temperature. When the temperature reached 700 °C, the concentration of Pb and Cd in the particulate matter significantly increased. The ecological risk assessment showed that heavy metals in the sewage sludge had considerable ecological toxicity. When the pyrolysis temperature reached 700 °C, the ecological toxicity of those heavy metals enriched in the particulate matter decreased considerably.

## 1. Introduction

With the rapid urbanization of China, the production of sewage sludge has dramatically increased [1,2]. Various toxic and harmful substances in sewage sludge, such as parasite eggs, pathogenic microorganisms, and heavy metals, cause the pollution of soil and water bodies when directly discharged into the environment [3,4,5,6]. Sludge pyrolysis technology can not only realize the harmless disposal of sludge, but also produce sludge biochar and bio-oil [7,8,9,10,11,12]. However, the high humidity (~80%) in the sludge requires a large amount of heat to complete the drying and pyrolysis process. Recently, sludge-combined treatment technology has provided an effective way to dispose of sewage sludge [13,14,15]. The object of this technology is to use the waste heat from the plant flue gas to complete the sludge drying and pyrolysis process, without requiring extra heat sources [16]. Although many waste heat systems exist in cement and power plants across China, the particulate matter (PM) of sludge pyrolysis could be introduced into the flue gas, which is difficult to remove with the original separation equipment [17,18,19].

The PM of sludge pyrolysis production has attracted widespread attention in recent years under the background of foggy weather. PM is also considered to be a carrier of some toxic substances, especially heavy metals. Most heavy metals (such as Pb, Cd, and Cr) are harmful to human health [20,21,22,23]. Ko et al. reported the characterizations of particulate matter formed during sewage sludge pyrolysis [24,25]. Cheng et al. investigated the mass balance and distribution of heavy metals in sewage sludge in step grate and fluidized bed incinerator [26]. Zha et al. noted that more attention should be paid to the potential risk of PM and heavy metal emissions in a fluidized bed furnace during sludge incineration [27]. The formation mechanism of the PM in municipal sewage sludge, treated by an indoor simulated cement kiln, was also studied by Yang et al. [28]. We also prepared biochar in different temperatures and found that PM was released into the air with the flue gas [29]. In sludge pyrolysis technology, the enrichment and morphological changes of heavy metals in PM are still not fully understood. If blindly discharged into the atmosphere, it will cause irreversible harm to the environment.

The main purpose of this work is to explore the effect of the pyrolysis temperature on the chemical morphology of heavy metals in PM, and to evaluate the potential environmental risks of heavy metals.

## 2. Materials and Methods

### 2.1. Pretreatment of the Sewage Sludge Samples

The sewage sludge used in this study was taken from the dehydration workshop of a municipal sewage treatment plant in Xi’an city. The sludge was dried naturally, then ground and sifted through a 100-mesh sieve. Ultimate analysis and proximate analysis of sludge samples are shown in Table 1, and heavy metal content and ash analysis are shown in Table 2.

### 2.2. Sludge Pyrolysis Devices and Collection of PM

**Figure 1:**
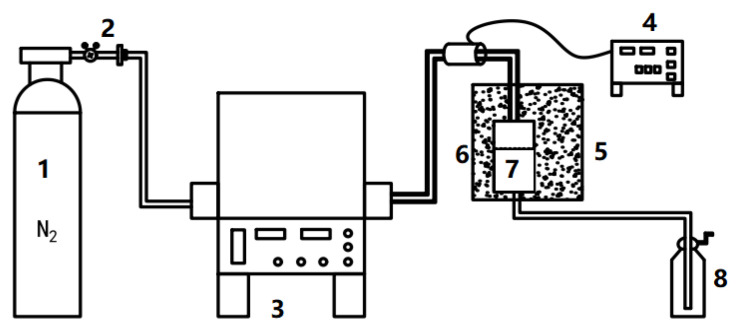
Sludge pyrolysis and PM collection device. 1: Nitrogen, 2: Rotor flow, 3: Tube furnace, 4: Heating tube, 5: Sand bath, 6: PM sampler, 7: Quartz filter, 8: Wash gas bottles.

The self-made sludge pyrolysis and PM collection device is exhibited in Figure 1. Sludge pyrolysis was completed in a tubular furnace (Quanshuo Electric Furnace Co., LTD, Shanghai, China). The PM produced by the pyrolysis process was blown into the PM collector. A quartz filter membrane (Necheng Technology Co., LTD, Suzhou, China) was placed in the PM collector to collect PM samples. To prevent condensation of volatiles, the PM collector was heated with a sand bath at a temperature of 300 °C. A heating sleeve (Xinming Technology Co., LTD, Taizhou, China) was used to keep the pipeline temperature at 300 °C to prevent deposition with volatile condensation. The PM mass was obtained, with the weight difference of PM mass, by weighing the quartz filter membranes before and after the pyrolysis experiment.

Sludge samples with a mass of 20 g each time were add to crucible. First, nitrogen was injected at a rate of 1 L/min for 10 min to sweep the air in the furnace. Then, the tube furnace was heated from room temperature to the end-point temperature at 300, 400, 500, 600, and 700 °C with a heating rate of 10 °C/min, and kept for 60 min.

### 2.3. Testing of PM Characteristics

The elemental contents of C, H, N and S in sewage sludge were determined using an organic element analyzer (Vario EL III, Hanau, Germany). Sludge was analyzed by an automated industrial analyzer (SDTGA5000, Sundy, Changsha, China). Ash component of the sewage sludge was determined using an X-ray fluorescence spectrometer (S4 PIONEER, Bruker, Karlsruhe, Germany). A thermogravimetric analyzer (TGA-50H, Shimadzu, Nagoya, Japan) was used to analyze the sludge. The blowing rate of N_2_ in thermogravimetric analysis was 40 mL/min, and heated from 50 °C to 1200 °C with a heating rate of 10 °C/min. The apparent morphology of the sample was analyzed by field emission scanning electron microscope (JSM-7610F, JEOL, Tokyo, Japan), and the elemental composition of the sample point was recorded by EDS analysis.

### 2.4. Analysis of Heavy Metals

#### 2.4.1. Concentrations and Chemical Forms of Heavy Metals

According to the type and content of heavy metals in sludge, the content and morphology of six heavy metals (Cu, Zn, Pb, Cd, Cr, and Ni) in PM samples were determined. The chemical morphology of heavy metals was analyzed using the three-step sequential extraction method (as specified and modified by the Community Bureau of Reference (BCR), Commission of the European Communities), as shown in Table 3. The total content of heavy metals was determined by the U.S. Environmental Protection Agency Method 3050B (US EPA 3050B) [30]. The heavy metal contents in the solutions were determined using an inductively coupled plasma-optical emission spectrometer (ICP-OES, 715-ES, Varian, Palo Alto, CA, USA).

#### 2.4.2. Ecological Risk Index (RI) of Heavy Metals

Based on the total concentration, quantity, toxicity, and sensitivity of heavy metals, Hakanson [31] evaluated the potential risk of heavy metal contamination in the samples. Using this calculation method, the ecological risk index (*RI*) can be calculated as follows:

(1) $$C_{f}=C_{i}/C_{n}$$

(2) $$E_{r}=T_{r}\cdot C_{f}$$

(3) $$RI=\sum E_{r}$$

where *C_f_* is the pollution factor of an individual heavy metal, *C_i_* represents the sum of this metal as measured in the (F1 + F2 + F3) section, and *C_n_* represents the score of the same metal in F4. *T_r_* represents the toxicity reaction factor of a specific heavy metal, and the values of each heavy metal are Zn (1), Cu (5), Cr (2), Ni (6), Pb (5) and Cd (30). *E_r_* is an ecological risk factor for a single heavy metal, multiplied by *T_r_* and *C_f_*. The sum of *E_r_* for all heavy metals in the sample is the ecological risk index (*RI*).

### 2.5. Statistical Analyses

All experiments were conducted in triplicate, and the results were expressed as only the mean values ± SD (standard deviation). The SPSS 22.0 statistical package (IBM, Armonk, NY, USA) was used for statistical analysis of the data. The least significant difference (LSD) method was used to analyze the deviation level of each average value, and the significant level was *p* < 0.05.

## 3. Results and Discussion

### 3.1. Analysis of Pyrolysis and PM Generation of Sewage Sludge

**Figure 2:**
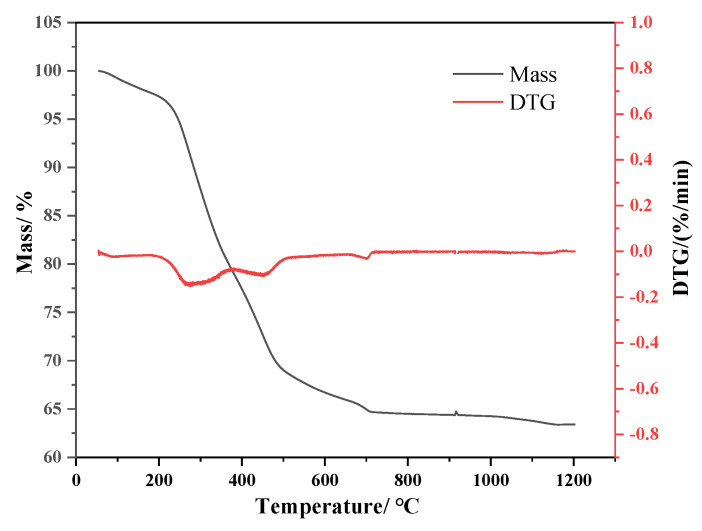
TGA and DTG curves during sludge pyrolysis.

Figure 2 shows the thermogravimetric (TG) curve and the weight loss rate (DTG) curve of sewage sludge. The sludge pyrolysis process can be divided into three stages. The first stage is the water evaporation stage (room temperature ~200 °C). The second stage is the stage of volatilization (200–500 °C). There are two similar peaks at this stage, as the organic matter in the sludge consists of proteins, aliphatic compounds, and sugars. With different bond energy, the temperature range of the bond breaks is also different. It can be inferred that the aliphatic compounds began to pyrolyze around 200 °C, the proteins began to pyrolyze around 300 °C, and the saccharides began to pyrolyze at about 390 °C. The third stage is the decomposition stage of the semi-coke (500–700 °C), which is mainly the heavy cyclization and coking reaction of the aromatic substances and unsaturated hydrocarbons.

**Figure 3:**
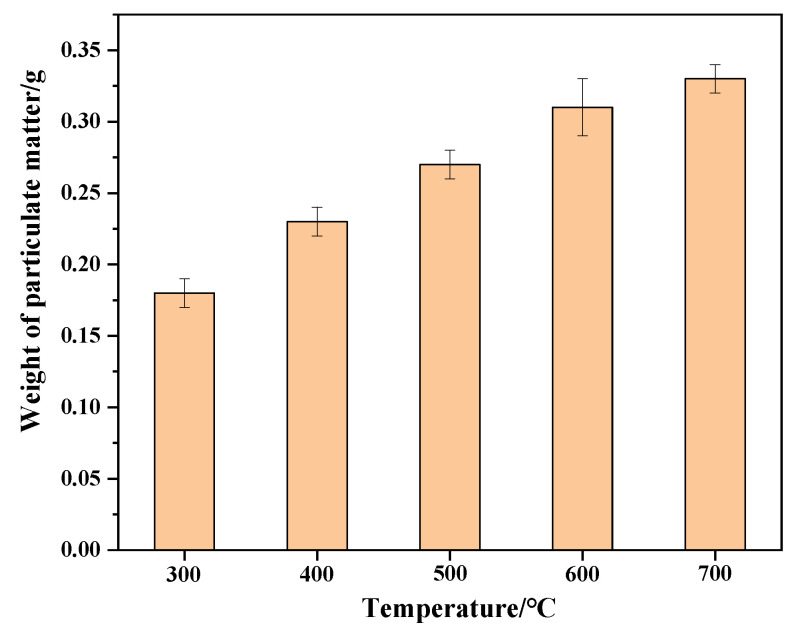
Mass of PM at different pyrolysis temperatures.

The change in sludge pyrolysis PM weight with the pyrolysis temperature is shown in Figure 3. It was obvious that the PM mass increased with the temperature. When the temperature was higher than 500 °C, the increase trend slowed down. Combined with the DTG curve analysis, the change process of the PM weight was mostly affected by the change trend of the pyrolysis gas phase products.

### 3.2. Scanning Electron Microscopy (SEM) Analysis

**Figure 4:**
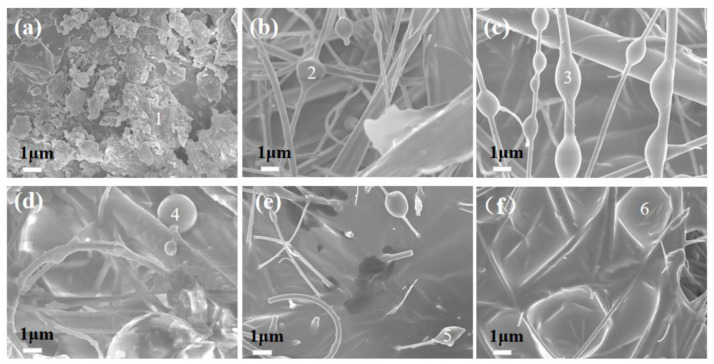
SEM images of PM collected with different temperatures. (**a**) SS, (**b**) PM-300, (**c**) PM-400, (**d**) PM-500, (**e**) PM-600, (**f**) PM-700. SS, sewage sludge. PM-X, PM derived from sewage sludge pyrolysis at X (°C) temperature.

Figure 4 shows a series of SEM images of the sewage sludge and PM, captured at different pyrolysis temperatures. As demonstrated in Figure 4a, a large number of irregular particles within several microns are attached to the surface of the sewage sludge. Figure 4b–f shows that the gas product condenses infiltrate into the spherical particles on the quartz filter membrane and increases with the temperature after pyrolysis. Non-spherical particles are found in Figure 4e,f.

The chemical elements of the selected points in Figure 4 are shown in Table 4. As demonstrated in Table 4, the particles attached to the surface of the sludge samples are mainly inorganic particles with Ca elements. When the pyrolysis temperature is 300–500 °C, the PM collected on the quartz filter membrane are mainly C element. That is due to the re-condensation of the organic matter on the quartz filter membrane. When the pyrolysis temperature is 600–700 °C, the C element content of the PM on the quartz filter membrane decreases and the Si element content increases, which indicates that the inorganic particles are captured by the quartz filter membrane after volatilization during pyrolysis.

### 3.3. Heavy Metal Analysis

#### 3.3.1. Content of Heavy Metals in PM

Table 5 shows the content of heavy metals in the PM. As shown in the table, Ni was not detected in the PM, indicating that the Ni of the sludge was not volatile. Other heavy metals were all higher in the PM than in the sludge. The heavy metals in the PM increase with the pyrolysis temperature, indicating the enrichment of heavy metals in PM. Compared with the heavy metal content of the PM at 600 °C, the content of Pb and Cd increased by 78.55% and 130.32% at 700 °C, respectively, while the content of heavy metals Cr, Cu, Ni, and Zn uniformly increased with the increase in pyrolysis temperature.

#### 3.3.2. Chemical Morphology Analysis of Heavy Metals in PM

The heavy metals in exchangeable and acid-soluble states (F1) and reducible states (F2) obtained by BCR sequential extraction can be directly utilized as bioactive heavy metals, which are easily leached. Heavy metals in oxidizable states (F3) are potentially bioactive heavy metals, which tend to degrade and leach in strong acids or in oxidation environments. However, heavy metals in residual states (F4) are non-biovalid heavy metals, which cannot be leached and degraded.

**Figure 5:**
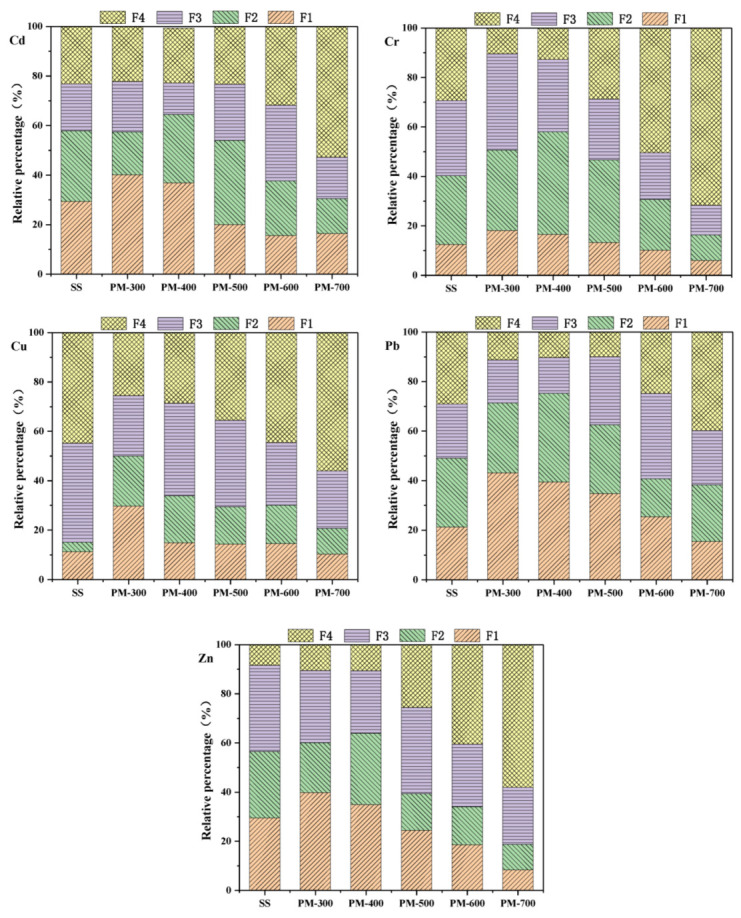
Chemical forms of heavy metals in sewage sludge and PM. F1, acid soluble and exchangeable states; F2, reducible fraction; F3, oxidizable fraction; F4, residual fraction.

Figure 5 shows the percentage of five heavy metals in the four chemical forms of sewage sludge obtained using the BCR method. The ratios of heavy metals (Zn, Cd, and Pb) in exchangeable and acid soluble, reducible, and oxidable (F1 + F2 + F3) sewage sludge were 91.62%, 76.85%, and 70.95%, respectively, indicating high potential ecological risks for sludge when directly discharged into the environment. With increased pyrolysis temperature, the residue (F4) content of the heavy metals (Zn, Cd, and Pb) in the PM gradually increased; when the pyrolysis temperature was 700 °C, the amounts reached 58.02%, 52.75%, and 39.77% respectively. This shows that the form of heavy metals volatilized to PM changes, forming a more stable residue state. The heavy metals Cu and Cr mainly exist in oxidizable (F3) and residue states (F4). In the PM from pyrolysis, the exchangeable and acid-soluble states (F1) and the reducible states (F2) rapidly decrease or even disappear. Overall, as the pyrolysis temperature rises, the five heavy metals in the PM move towards a more stable morphology. This shows that the pyrolysis temperature is an important factor in the transformation of heavy metals in the PM to a more stable state.

#### 3.3.3. Ecological Risk Assessment of Heavy Metals

**Figure 6:**
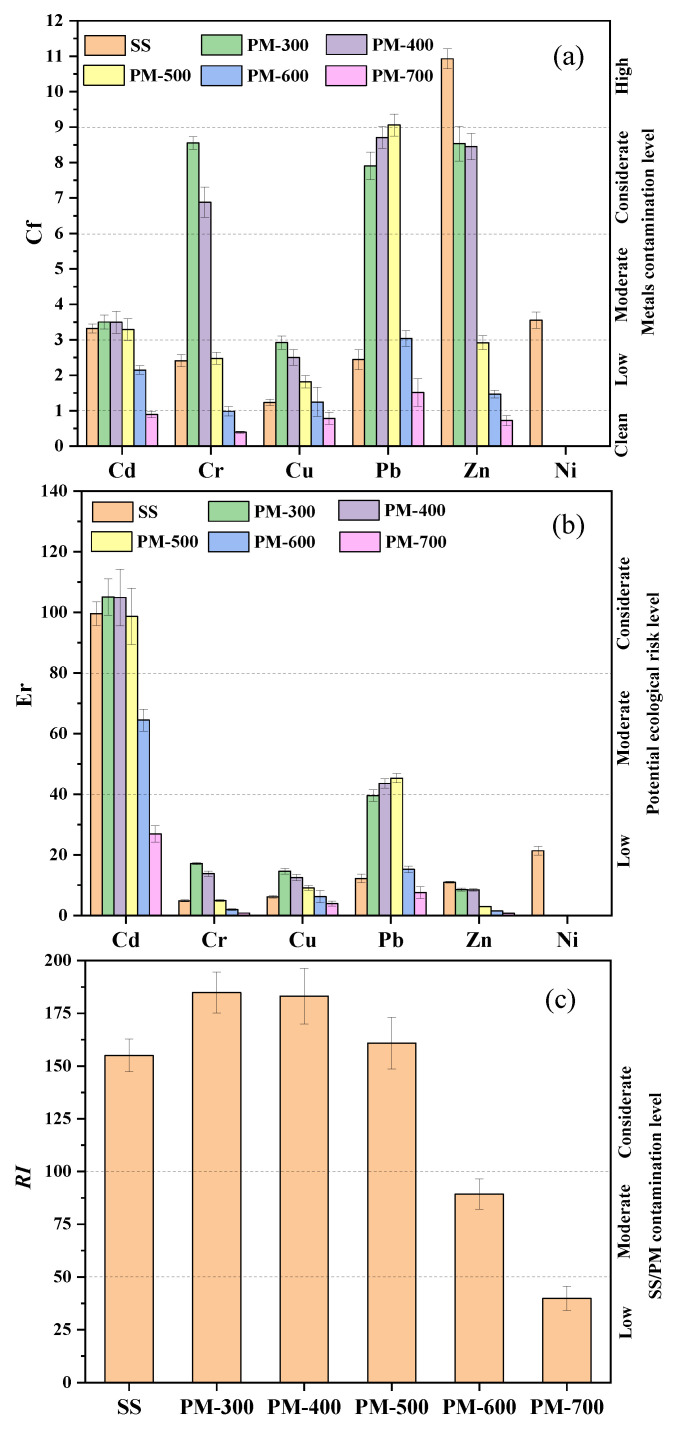
Ecological risk assessment of the heavy metals in the SS and PM. (**a**) contamination factor of the individual heavy metal; (**b**) potential ecological risk factor for the individual heavy metal; (**c**) the sum of the potential ecological risk index (*E_r_*) of each heavy metal. PM-X, PM derived from sewage sludge pyrolysis at X (°C) temperature.

Figure 6 shows the calculated values of *C_f_*, *E_r_*, and *RI*. As depicted in Figure 6a, the *C_f_* values of heavy metals, Cd, Cr, Cu, and Pb obtained at 300 °C of PM, were higher than the sludge samples. It was demonstrated that pyrolysis at 300 °C favors the transfer of unstable states of heavy metals to PM. With the increase in pyrolysis temperature, the *C_f_* of heavy metals (Cd, Cr, and Cu) significantly decreased in PM. After the pyrolysis temperature reached 700 °C, it posed no ecological risk. The *C_f_* value of the Pb increased with the pyrolysis temperature and then decreased, the highest value appearing at 500 °C. The *C_f_* value of the Zn in the PM was lower than that of the sludge sample and decreased with the pyrolysis temperature, the minimum value appearing at 700 °C. Increasing the pyrolysis temperature can significantly reduce the *C_f_* value, and each heavy metal reached a relative minimum at 700 °C.

The *E_r_* demonstrates that the potential environmental impact of Cd and Pd is high. The potential ecological risk of heavy metals in sludge samples and in PM increases with increasing pyrolysis temperature and then decreases. When the pyrolysis temperature is 700 °C, the RI value is the lowest and low-risk status is achieved.

## 4. Summary

In this paper, the characteristics of PM produced at different pyrolysis temperatures and the environmental risk assessment of heavy metals were studied. The results show that the PM produced at 300–500 °C, attributed to the volatilization of organic matter. When the pyrolysis temperature is 600–700 °C, the C element content of the PM on the quartz filter membrane decreases, and the Si element content increases, indicating that inorganic particles are captured by the quartz filter membrane after volatilization during pyrolysis. Heavy metals in the PM increase with the increasing pyrolysis temperature, indicating that heavy metals were enriched in the PM. With the increase in pyrolysis temperature, the proportion of heavy metal residue in the PM increases, confirming that the increasing pyrolysis temperature can promote the transformation of heavy metals into a more stable state. The ecological risk assessment showed that the heavy metals in sewage sludge had considerable ecological toxicity. When the pyrolysis temperature reached 700 °C, the ecological toxicity of the heavy metals in the PM decreased to low-risk.

## Figures and Tables

**Table 1:** Ultimate analysis and proximate analysis of the sewage sludge.

Ultimate Analysis (wt%)					Proximate Analysis (wt%)			
C	H	N	S	O	M	A	V	FC
31.77 ± 0.04	4.30 ± 0.36	2.78 ± 0.06	0.61 ± 0.01	21.69 ± 0.53	3.04 ± 0.05	38.85 ± 0.10	53.38 ± 0.23	4.73

By difference, O (%) = 100-C-H-N-S-Ash, Fixed Carbon (%) = 100-Ash-Volatile-Moisture.

**Table 2:** Heavy metals and ash component of the sewage sludge.

Heavy Metal (mg·kg^−1^)						Ash Component (%)						
Cd	Cr	Cu	Ni	Pb	Zn	SiO_2_	Al_2_O_3_	Fe_2_O_3_	CaO	MgO	K_2_O	Na_2_O
7.05 ± 0.01	124.15 ± 0.86	134.70 ± 0.88	14.29 ± 0.06	121.78 ± 0.23	1341.73 ± 6.25	47.01 ± 0.16	14.29 ± 0.13	9.55 ± 0.08	6.98 ± 0.10	3.06 ± 0.02	2.82 ± 0.09	1.72 ± 0.01

**Table 3:** Modified BCR three-step sequential extraction procedure.

Fraction		Extraction Reagents	Extraction Conditions
F1:	acid soluble and exchangeable states	20 mL, 0.01 M HAc	Oscillating, 22 ± 5 °C, 16 h
F2:	reducible states	20 mL, 0.50 M NH_2_OH·HCl	Oscillating, 22 ± 5 °C, 16 h
F3:	oxidisable states	First, 5 mL, 30% (*v*/*v*) H_2_O_2_, Next, 5 mL, 30% (*v*/*v*) H_2_O_2_, Last, 25 mL, 1.0 M CH_2_COONH_4_	First, water bath, 85 ± 5 °C, 1 h, Next, water bath, 85 ± 5 °C, 1 h, Last, Oscillating, 22 ± 5 °C, 16 h
F4:	residual states	5 mL, HNO_3_, 3 mL, HClO_4_, 5 mL, HF	Heating of heating plate

**Table 4:** Chemical compositions determined by EDS of crystals.

Position	C/wt%	N/wt%	O/wt%	Al/wt%	Si/wt%	S/wt%	Ca/wt%	Cu/wt%	Zn/wt%
a-1	14.53 ± 0.27	1.56 ± 0.20	8.59 ± 0.37	3.87 ± 0.17	9.98 ± 0.18	1.07 ± 0.03	48.96 ± 0.48	0.13 ± 0.01	0.49 ± 0.02
b-2	65.76 ± 0.46	1.17 ± 0.03	18.09 ± 0.18	0.68 ± 0.01	7.12 ± 0.09	0.54 ± 0.02	1.85 ± 0.05	0.01 ± 0.01	0.74 ± 0.02
c-3	63.26 ± 0.10	2.05 ± 0.10	17.37 ± 0.05	0.87 ± 0.06	9.97 ± 0.13	1.11 ± 0.03	ND	0.01 ± 0.00	0.92 ± 0.03
d-4	68.92 ± 0.09	3.32 ± 0.02	17.62 ± 0.05	0.68 ± 0.01	6.18 ± 0.03	0.45 ± 0.03	0.30 ± 0.02	0.02 ± 0.01	0.94 ± 0.02
e-5	40.07 ± 0.16	1.22 ± 0.01	21.90 ± 0.67	2.09 ± 0.11	19.48 ± 0.11	1.61 ± 0.03	3.11 ± 0.04	0.02 ± 0.01	1.87 ± 0.03
f-6	32.07 ± 0.19	0.64 ± 0.04	19.36 ± 0.08	2.80 ± 0.12	32.50 ± 0.10	0.33 ± 0.04	4.29 ± 0.10	0.03 ± 0.01	1.92 ± 0.03

ND: Not detected (<0.01 wt%).

**Table 5:** Content of heavy metals in PM.

Samples	Heavy Metals (mg·kg^−1^)					
	Cd	Cr	Cu	Ni	Pb	Zn
PM-300	16.28 ± 0.61	149.15 ± 0.77	110.15 ± 3.82	ND	137.38 ± 2.94	7447.94 ± 40.56
PM-400	17.61 ± 0.38	166.51 ± 1.20	150.49 ± 3.04	ND	139.50 ± 2.03	8412.14 ± 150.31
PM-500	19.22 ± 0.46	181.88 ± 0.73	154.51 ± 4.45	ND	156.60 ± 1.55	11,011.31 ± 257.27
PM-600	25.03 ± 1.39	204.38 ± 1.43	175.18 ± 1.07	ND	187.90 ± 10.34	15,202.34 ± 165.55
PM-700	57.65 ± 2.89	236.87 ± 1.20	190.15 ± 3.31	ND	335.50 ± 18.16	16,665.07 ± 261.35

ND: Not detected (<0.01 mg/kg). PM-X, PM derived from sewage sludge pyrolysis at X (°C) temperature.

## Data Availability

The data presented in this study are available on request from the corresponding author.

## References

[1] Xia Y., Tang Y., Shih K., Li B. (2020). Enhanced phosphorus availability and heavy metal removal by chlorination during sewage sludge pyrolysis. J. Hazard. Mater..

[2] Fan H., Lv M., Wang X., Xiao J., Mi X., Jia L. (2020). Effect of Cr on the Mineral Structure and Composition of Cement Clinker and Its Solidification Behavior. Materials.

[3] Thomsen T.P., Sárossy Z., Ahrenfeldt J., Henriksen U.B., Frandsen F.J., Müller-Stöver D. (2017). Changes imposed by pyrolysis, thermal gasification and incineration on composition and phosphorus fertilizer quality of municipal sewage sludge. J. Environ. Manag..

[4] Xu Q., Tang S., Wang J., Ko J.H. (2018). Pyrolysis kinetics of sewage sludge and its biochar characteristics. Process. Saf. Environ. Prot..

[5] Jin J., Li Y., Zhang J., Wu S., Cao Y., Liang P., Zhang J., Wong M.H., Wang M., Shan S. (2016). Influence of pyrolysis temperature on properties and environmental safety of heavy metals in biochars derived from municipal sewage sludge. J. Hazard. Mater..

[6] Jin H., Renato O., Arazo J.G. (2014). Leaching of heavy metals from fast pyrolysis residues produced from different particle sizes of sewage sludge. J. Anal. Appl. Pyrolysis.

[7] Fonts I., Gea G., Azuara M., Ábrego J., Arauzo J. (2012). Sewage sludge pyrolysis for liquid production: A review. Renew. Sustain. Energy Rev..

[8] Méndez A., Paz-Ferreiro J., Araujo F., Gascó G. (2014). Biochar from pyrolysis of deinking paper sludge and its use in the treatment of a nickel polluted soil. J. Anal. Appl. Pyrolysis.

[9] Wang X., Li C., Li Z., Yu G., Wang Y. (2019). Effect of pyrolysis temperature on characteristics, chemical speciation and risk evaluation of heavy metals in biochar derived from textile dyeing sludge. Ecotoxicol. Environ. Saf..

[10] Devi P., Saroha A.K. (2014). Risk analysis of pyrolyzed biochar made from paper mill effluent treatment plant sludge for bioavailability and eco-toxicity of heavy metals. Bioresour. Technol..

[11] Zheng H., Wang Z., Deng X., Zhao J., Luo Y., Novak J., Herbert S., Xing B. (2013). Characteristics and nutrient values of biochars produced from giant reed at different temperatures. Bioresour. Technol..

[12] Yuan X., Leng L., Huang H.-J., Chen X., Wang H., Xiao Z., Zhai Y., Chen H., Zeng G. (2015). Speciation and environmental risk assessment of heavy metal in bio-oil from liquefaction/pyrolysis of sewage sludge. Chemosphere.

[13] Zhai Y., Chen H., Xu B., Xiang B., Chen Z., Li C., Zeng G. (2014). Influence of sewage sludge-based activated carbon and temperature on the liquefaction of sewage sludge: Yield and composition of bio-oil, immobilization and risk assessment of heavy metals. Bioresour. Technol..

[14] Sánchez M., Menéndez J., Domínguez A., Pis J., Martínez O., Calvo L., Bernad P. (2009). Effect of pyrolysis temperature on the composition of the oils obtained from sewage sludge. Biomass Bioenergy.

[15] Khanmohammadi Z., Afyuni M., Mosaddeghi M.R. (2015). Effect of pyrolysis temperature on chemical and physical properties of sewage sludge biochar. Waste Manag. Res..

[16] Liu T., Liu Z., Zheng Q., Lang Q., Xia Y., Peng N., Gai C. (2018). Effect of hydrothermal carbonization on migration and environmental risk of heavy metals in sewage sludge during pyrolysis. Bioresour. Technol..

[17] Schuhmacher M., Nadal M., Domingo J.L. (2009). Environmental monitoring of PCDD/Fs and metals in the vicinity of a cement plant after using sewage sludge as a secondary fuel. Chemosphere.

[18] Huang H.-J., Yuan X.-Z. (2016). The migration and transformation behaviors of heavy metals during the hydrothermal treatment of sewage sludge. Bioresour. Technol..

[19] Song X., Xue X., Chen D., He P., Dai X. (2014). Application of biochar from sewage sludge to plant cultivation: Influence of pyrolysis temperature and biochar-to-soil ratio on yield and heavy metal accumulation. Chemosphere.

[20] Collier S., Zhou S., Kuwayama T., Forestieri S., Brady J., Zhang M., Kleeman M., Cappa C., Bertram T., Zhang Q. (2015). Organic PM Emissions from Vehicles: Composition, O/C Ratio, and Dependence on PM Concentration. Aerosol Sci. Technol..

[21] Han Y., Hwang G., Kim D., Park S., Kim H. (2015). Porous Ca-based bead sorbents for simultaneous removal of SO2, fine particulate matters, and heavy metals from pilot plant sewage sludge incineration. J. Hazard. Mater..

[22] Yang Z., Zhang Y., Liu L., Wang X., Zhang Z. (2016). Environmental investigation on co-combustion of sewage sludge and coal gangue: SO_2_, NOx and trace elements emissions. Waste Manag..

[23] Wang G., Zhang R., Gomez M.E., Yang L., Zamora M.L., Hu M., Lin Y., Peng J., Guo S., Meng J. (2016). Persistent sulfate formation from London Fog to Chinese haze. Proc. Natl. Acad. Sci. USA.

[24] Ko J.H., Wang J., Xu Q. (2018). Characterization of particulate matter formed during sewage sludge pyrolysis. Fuel.

[25] Ko J.H., Wang J., Xu Q. (2018). Impact of pyrolysis conditions on polycyclic aromatic hydrocarbons (PAHs) formation in particulate matter (PM) during sewage sludge pyrolysis. Chemosphere.

[26] Cheng Y., Oleszek S., Shiota K., Oshita K., Takaoka M. (2020). Comparison of sewage sludge mono-incinerators: Mass balance and distribution of heavy metals in step grate and fluidized bed incinerators. Waste Manag..

[27] Zha J., Huang Y., Clough P.T., Dong L., Xu L., Liu L., Zhu Z., Yu M. (2020). Desulfurization using limestone during sludge incineration in a fluidized bed furnace: Increased risk of particulate matter and heavy metal emissions. Fuel.

[28] Yang Z., Wang C., Wang J., Liu L., Ge X., Zhang Z. (2019). Investigation of formation mechanism of particulate matter in a laboratory-scale simulated cement kiln co-processing municipal sewage sludge. J. Clean. Prod..

[29] Li B., Ding S., Fan H., Ren Y. (2021). Experimental Investigation into the Effect of Pyrolysis on Chemical Forms of Heavy Metals in Sewage Sludge Biochar (SSB), with Brief Ecological Risk Assessment. Materials.

[30] (1996). Method 3050B: Acid Digestion of Sediments, Sludges, and Soils.

[31] Hakanson L. (1980). An ecological risk index for aquatic pollution control.a sedimentological approach. Water Res..

